# Pituitary metastasis from renal cell carcinoma presenting with significant hyperprolactinemia, case report

**DOI:** 10.1002/ccr3.7808

**Published:** 2023-08-25

**Authors:** Ali Mazar‐Atabaki, Omid Mohamadzadeh, Seyed Mousa Sadrehosseini, Azin Tabari, Mehdi Zeinalizadeh

**Affiliations:** ^1^ Brain and Spinal Injury Research Center, Pituitary Clinic Imam Khomeini Hospital Complex, Tehran University of Medical Sciences Tehran Iran; ^2^ Department of Neurological Surgery, Imam Khomeini Hospital Complex Tehran University of Medical Sciences Tehran Iran; ^3^ Department of Otolaryngology‐Head and Neck Surgery, Imam Khomeini Hospital Complex Tehran University of Medical Sciences Tehran Iran; ^4^ Brain and Spinal Injury Research Center, Pituitary Clinic Neuroscience Institute, Tehran University of Medical Sciences Tehran Iran

**Keywords:** endonasal approach, hyperprolactinemia, pituitary metastasis, renal cell carcinoma

## Abstract

**Key Clinical Message:**

Kidney metastasis to the pituitary gland can cause hyperprolactinemia even above 250 ng/mL. Although the treatment of metastasis is palliative, surgical decompression could play a major role in the recovery of symptoms and improve quality of life. Pituitary metastasis should be considered in the evaluation of an unusual pituitary mass.

**Abstract:**

Pituitary tumors are frequently encountered in the neurosurgical setting. Although the majority of them are pituitary adenomas, rare entities encompass pituitary metastasis. They should be differentiated from pituitary adenomas because their management and prognosis are different. We report a 53‐year‐old female who complained of headache and had remarkable hyperprolactinemia (271.1 ng/mL). Having considered macroprolactinoma as the initial diagnosis, medical treatment was initiated with Cabergoline. Subsequently, the patient's vision deteriorated which prompted us to perform endoscopic endonasal transsphenoidal surgery. Histologic examination of the resected tumor revealed metastatic renal cell carcinoma. Main treatment for these subjects is palliative; and unlike the pituitary adenoma, the prognosis is unfortunately poor. Pituitary metastasis should be considered in the evaluation of an unusual pituitary mass associated with hyperprolactinemia.

## Graphical abstract

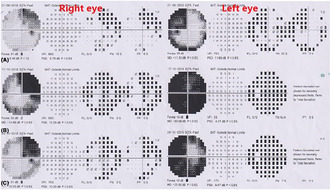

## 1 INTRODUCTION

Pituitary metastases, although rare, should be differentiated from pituitary adenomas or other pathologies in this region, because their treatment and prognosis vary widely. Systemic metastasis to the pituitary gland occurs in the late stages of the disease, but in 20%–30% of patients, could appear as the first manifestation of malignancy.^1^ Radiologic and laboratory findings could differentiate a pituitary metastasis from an adenoma, but they might be misleading. In this paper, we illustrate a case of pituitary metastasis as the primary clinical presentation of a renal cell carcinoma (RCC) associated with hyperprolactinemia of more than 200 ng/mL.

## 2 CASE REPORT

**FIGURE 1:**
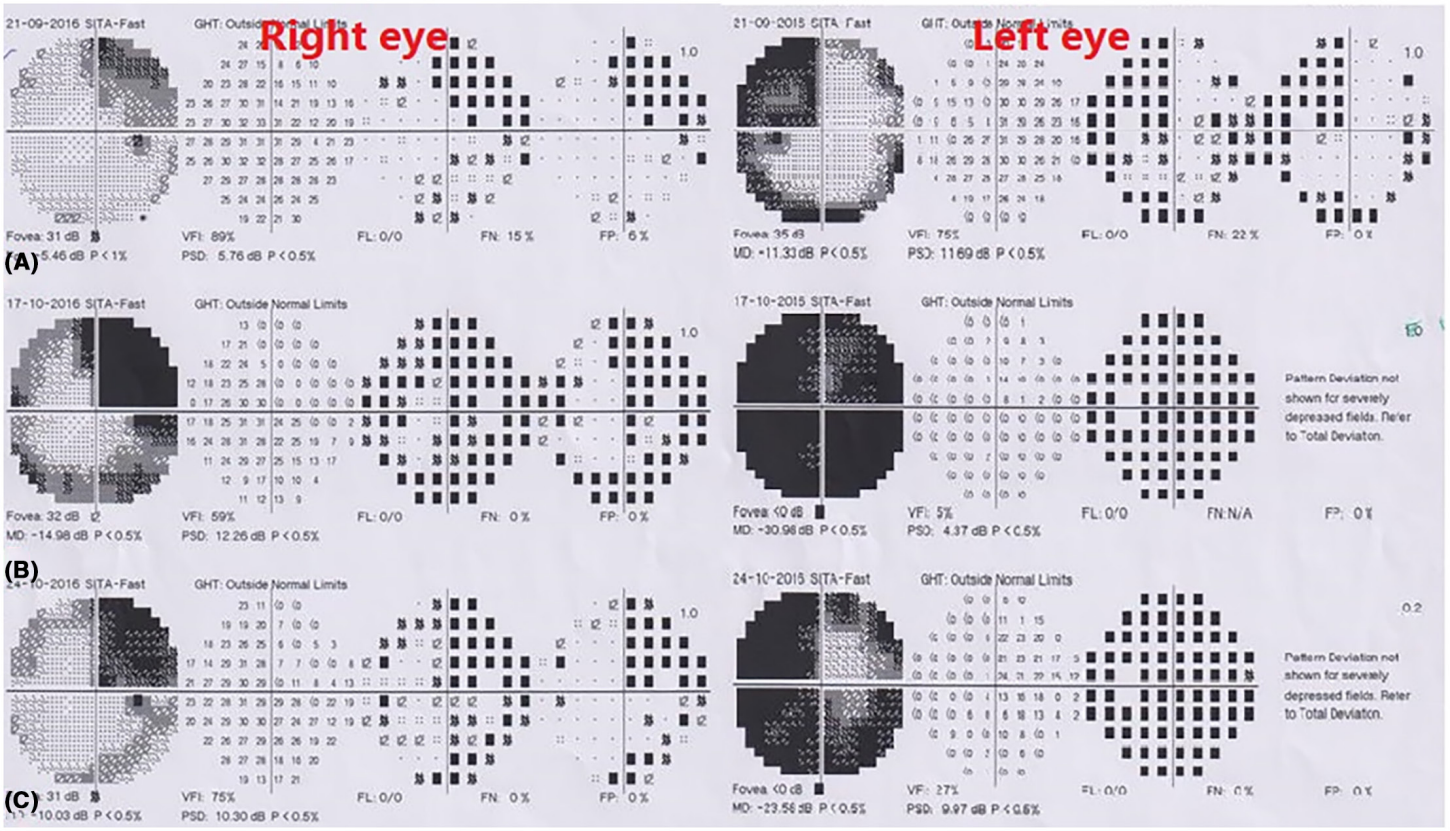
(A) Perimetry test before medical treatment, (B) Perimetry test just before endoscopic endonasal surgery. (C) Perimetry 4 days after surgery.

**FIGURE 2:**
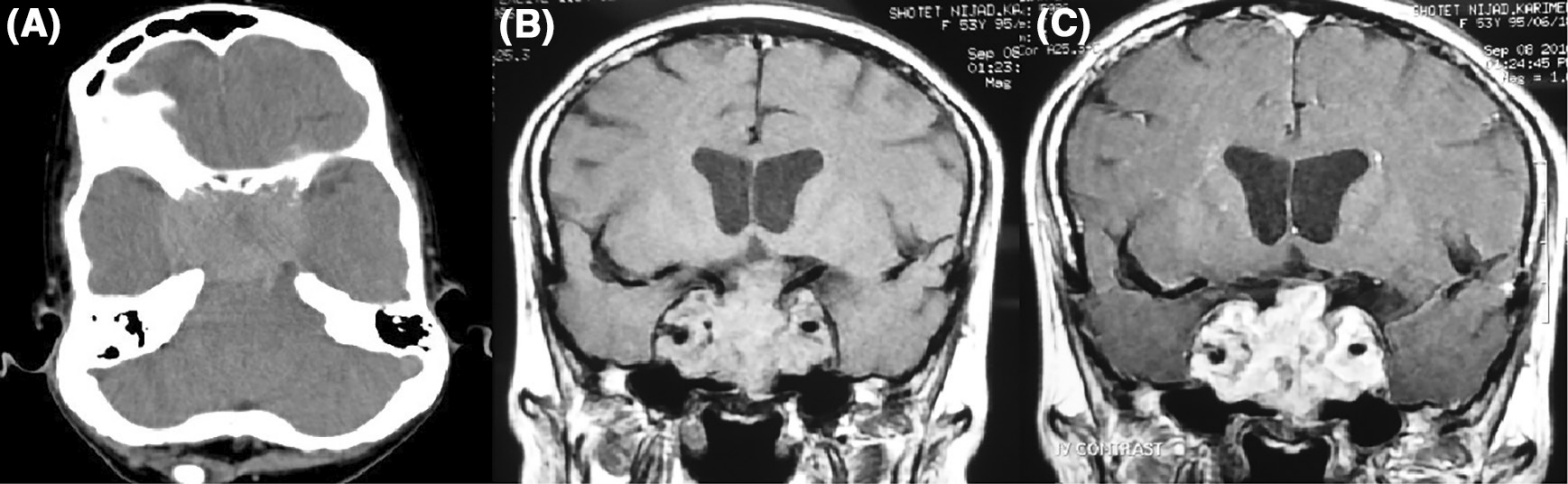
(A) Brain CT scan without contrast injection showing a hyperdense mass in the sellar/suprasellar region invading optic canals. (B) Coronal MRI views without gadolinium administration. (C) Coronal MRI with GD demonstrates a huge sellar tumor with suprasellar extension and bilateral cavernous sinus invasion.

**FIGURE 3:**
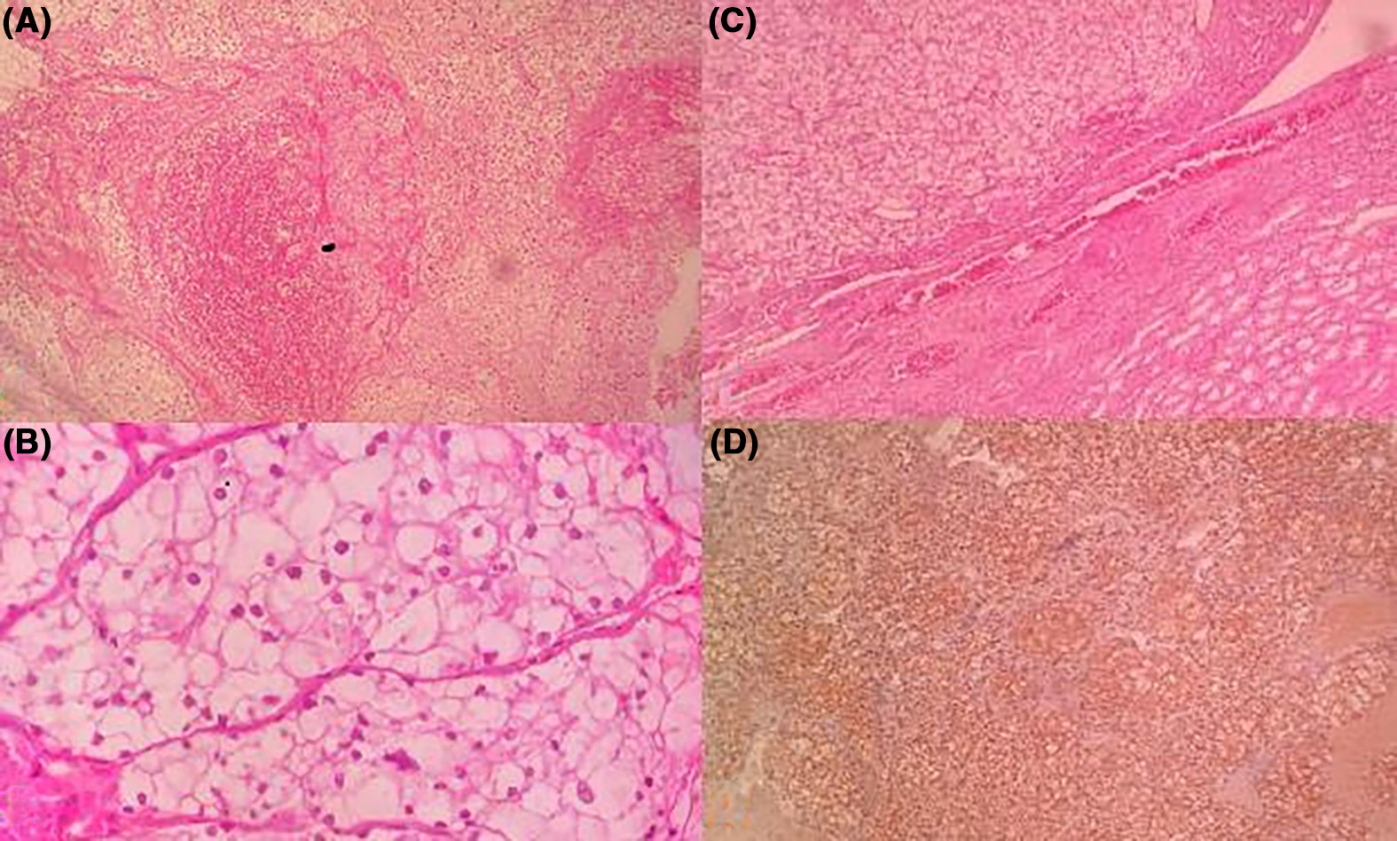
Low‐power (A) and high‐power (B) photomicrograph of sellar/suprasellar tumor shows nests of large cells, optically clear cytoplasm, and sharply outlines cell membrane in a highly vascularized stroma. Low‐power photomicrograph of renal tumor confirms clear cell type of renal cell carcinoma (C) that shows intense positive reaction to PRL in immunohistochemical examination (D).

**FIGURE 4:**
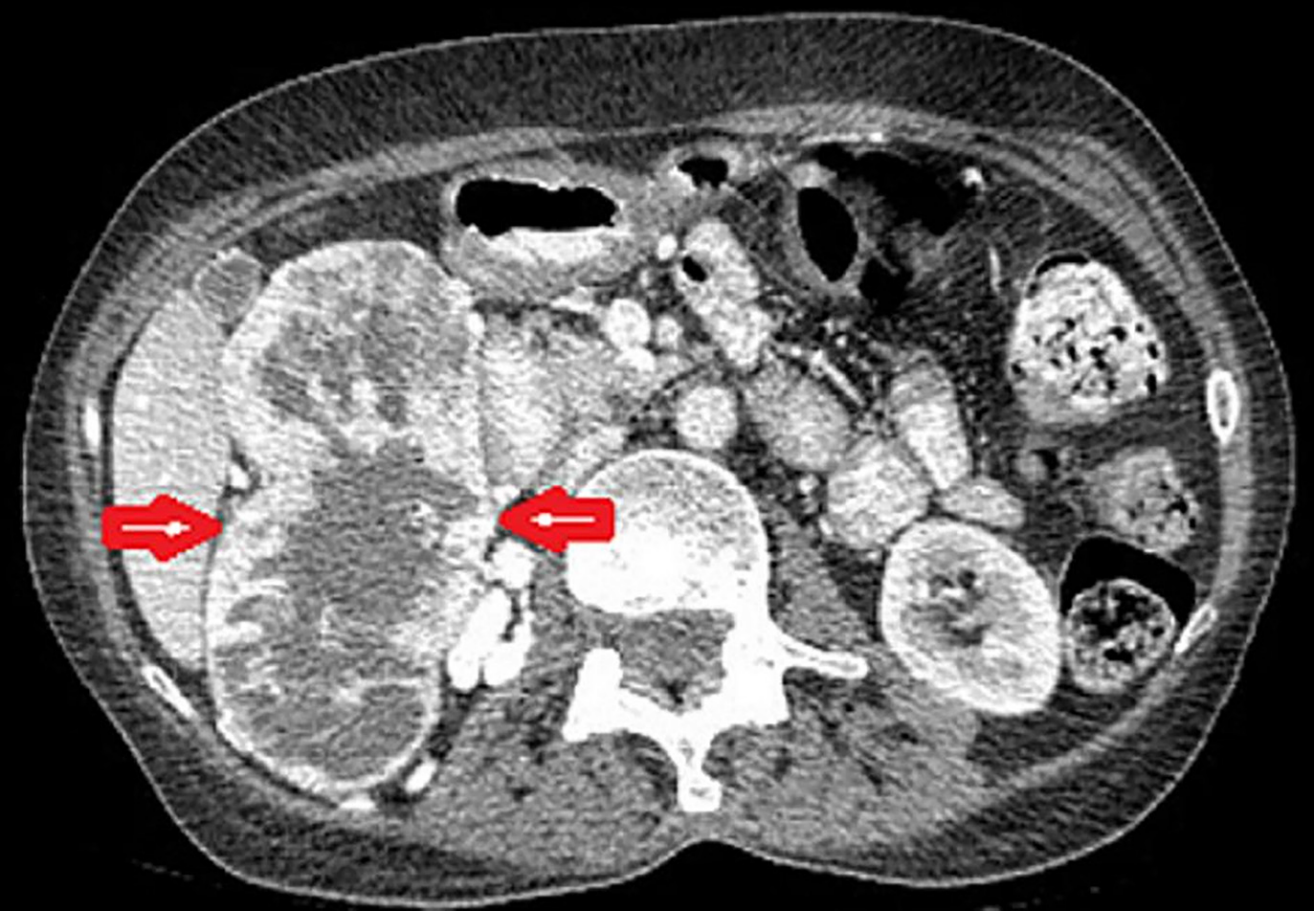
Abdominopelvic CT scan with intravenous contrast injection reveals right renal mass with heterogeneous enhancement.

A 53‐year‐old woman was referred to our pituitary clinic for evaluation of a giant pituitary mass. The patient complained of a headache 3 months ago that did not respond to medication and was getting worse. She was otherwise normal, except for being in her menopausal stage since 1 year ago, and had not noticed any visual impairment or change in her body weight. She had no complaints of galactorrhea, polyuria, or polydipsia. Physical examination showed mild bitemporal hemianopia with Visual field index (VFI) parameter in perimetry represent 89% and 75% in the right and left eyes respectively, with intact visual acuity that was confirmed by a subsequent perimetry test (Figure 1A). The rest of the examination was normal. Computed tomography (CT) scan revealed a huge hyperdense sellar mass (Figure 2A). Magnetic resonance imaging (MRI) revealed a 51 × 43 × 33 mm sellar mass with suprasellar extension and bilateral Knosp IV cavernous sinus invasion as well as a non‐homogenous and avid enhancement after gadolinium injection (Figure 2B; Figure 2C). Pituitary hormones profile revealed: TSH = 0.2 μIU/mL (0.5–4.5), T4 = 8.3 μg/dL (6.5–12), Cortisol = 2.3 μg/dL (6.2–20), LH = 0.9 mIU/mL (3.5–41), FSH = 2.8 mIU/mL (32–128), Prolactin = 174 ng/mL (4.79–23.3), GH = 1.2 ng/mL, and IGF‐1 = 95 ng/mL (53–278). Serum prolactin level was re‐checked with serum dilution and Poly Ethylene Glycol test that was 271.1 ng/mL. Macroprolactinoma was considered the first diagnosis, and medical treatment with Cabergoline increasing gradually to 0.5 mg three times a week along with corticosteroids was started. After 3 weeks, she was admitted to the emergency department with sudden left eye visual loss, nausea, and vomiting. Physical examination and perimetry showed the bilateral worsened visual field to 59% and 5% in the right and left eyes, respectively, and left eye visual acuity declined to 1/10 on the Snellen chart (Figure 1B). Laboratory data showed normal prolactin level (3 ng/mL) and hypocortisolism (3 μg/dL). CT scan and MRI showed no evidence of hemorrhage or any obvious changes in the size of the mass compared to previous images. The patient underwent endoscopic endonasal transsphenoidal surgery aimed at optic nerve decompression. Sella turcica floor was found to be eroded by the tumor. The tumor was firm, and highly vascular with profuse bleeding during the manipulation which necessitated an intraoperative blood transfusion. Hence, after optic nerve decompression, the operation was terminated. Visual acuity is not changed after 4 days of surgery, but VFI improved to 75% and 27% in the right and left eyes, respectively (Figure 1C). Histologic examination of the specimen showed nests of large cells, optically clear cytoplasm, and sharply outlines cell membrane in a highly vascularized stroma (Figure 3A,B). The immunohistochemical (IHC) study was positive for EMA, CK AE1/AE3, CD10, PRL, and Vimentin; and a metastatic RCC was confirmed. Consequently, an abdominopelvic CT scan revealed a 12.5 × 8 × 5 cm right renal mass (Figure 4) for which the patient underwent radical nephrectomy. The pathological study confirmed RCC, and interestingly, IHC study of the renal mass showed an intensely positive reaction for prolactin (Figure 3C,D). The patient underwent sellar radiotherapy but left eye vision was lost completely again due to the invasion of the tumor to the optic canal.

## 3 DISCUSSION

Metastatic tumors involving the pituitary gland are uncommon, and symptomatic pituitary metastasis of RCC is very rare.^2^ Although two‐thirds of pituitary metastases are from breast and lung cancer, metastases from almost all malignancies have been reported, however.^3^ Additionally, RCC is the primary cancer in only 2.6% of pituitary metastases. Because of direct arterial blood supply, pituitary metastases have a tendency toward the posterior pituitary lobe and present with signs of diabetes insipidus, although the anterior pituitary lobe could be involved with the local spread of the tumor. In the reported cases of metastatic RCC to the pituitary gland, hypopituitarism (80%) was reported as more common than diabetes insipidus (28%). Taken together, diabetes insipidus is the most frequent manifestation of pituitary metastases.^4^

Although hypophysis involvement by systemic cancers is uncommon, it should be considered in the differential diagnosis of a sellar mass, because it can change the management and prognosis of a pituitary tumor especially when the pertinent medical history is present. In 20%–30% of patients, metastasis to the pituitary gland is the first manifestation of a tumor of unknown origin.^1^ In these patients, the rapid course of the disease, ophthalmoplegia, diabetes insipidus, and headache should raise suspicion for pituitary metastasis.^5^ Pituitary metastases are usually asymptomatic and often present in disseminated cancer settings; so, characteristic clinical pituitary signs are usually overwhelmed by symptoms associated with primary systemic cancer or concomitant extra‐pituitary metastases.^6^ Based on our knowledge, our patient was the first case of an RCC who primarily presented with symptoms of pituitary metastasis associated with very high levels of serum prolactin.

For the precise evaluation of a pituitary mass, MRI is necessary. Although based on the MRI feature, pituitary metastasis cannot be readily distinguished from a pituitary adenoma or other lesions, some features could be helpful. In comparison with pituitary adenoma thickening of the pituitary stalk, erosion of bony element of sella turcica, loss of normal high signal intensity of posterior pituitary lobe, presence of flow voids in the tumor, and multiple lesions are in favor of pituitary metastasis.^1^, ^4^, ^5^ 18 fluorodeoxyglucose positron emission tomography highlighted sellar tumoral hypermetabolism, but it has revealed no significant difference in standardized uptake values between malignant and benign lesions.^7^

The special feature of this case is hyperprolactinemia. In the presence of a pituitary tumor together with hyperprolactinemia, most patients with prolactin levels higher than 250 ng/mL should be considered as cases of prolactinoma.^8^ In our patient, serum prolactin level was 271.1 ng/mL, a misleading level for the right diagnosis. Based on the literature, this level of hyperprolactinemia was the highest level in association with metastasis of RCC to the hypophysis.

According to the published data, in two historical cases have been reported to have hyperprolactinemia associated with RCC but serum prolactin levels less than 200 ng/mL. In both cases, serum prolactin levels returned to baseline levels after nephrectomy without any imaging of the pituitary region.^9^, ^10^ S.Basaria and his colleagues reported a 77‐year‐old woman with a hypophysial mass associated with RCC. The initial presentation was intermittent diplopia, and the preoperative prolactin level was 186 ng/mL. The patient underwent debulking endoscopic endonasal surgery, and the biopsy result was metastatic RCC.^11^ Other endocrinological abnormalities including elevated human chorionic gonadotropin (HCG) or adrenocorticotropic hormone (ACTH), Cushing's syndrome, and hyper/hypoglycemia can be seen as a paraneoplastic syndrome in renal cell carcinoma.^12^

Interestingly, in our case, the IHC study of the renal mass specimen was positive for the PRL marker same as that for the sellar tumor specimen. So, the renal cell tumor probably was also involved in prolactin secretion.

The best management of pituitary metastases has not been clearly determined. Surgical decompression could play a role in symptom palliation and probably improve quality of life. However, achieving gross total resection because of local invasiveness, high tumor vascularization, and cavernous sinus involvement as in our patient might be impossible. Other modalities of treatment include post‐surgical radiotherapy and stereotactic radiosurgery.

Unfortunately, the overall survival of these patients despite surgical tumor removal and radiation is poor with a median survival of 6–7 months.^13^

## 4 CONCLUSION

Pituitary metastasis should be considered in the evaluation of an unusual pituitary mass associated with hyperprolactinemia. The paraneoplastic syndrome of certain tumors, such as RCC, can be misleading in the diagnosis and treatment strategy of a pituitary mass. The overall prognosis in this group is generally poor and depends on the treatment of the underlying disease. Surgical treatment may help to improve the symptoms especially visual improvement and the quality of life.

## AUTHOR CONTRIBUTIONS

**Ali Mazar‐Atabaki:** Conceptualization; data curation; formal analysis; investigation; project administration; software; validation; writing – original draft. **Omid mohamadzadeh:** Data curation; methodology; writing – review and editing. **Seyed Mousa Sadrehosseini:** Conceptualization; supervision. **Azin Tabari:** Supervision; visualization. **Mehdi Zeinalizadeh:** Conceptualization; funding acquisition; resources; supervision.

## FUNDING INFORMATION

There was no source of funding for this study.

## CONFLICT OF INTEREST STATEMENT

The authors declare to have no conflicting interest.

## CONSENT

Written informed consent was obtained from the patient for publication of this case report and any accompanying images. A copy of the written consent is available for review by the Editor‐in‐Chief of this journal.

## Data Availability

Data that support the findings of this study are available from the corresponding author.

## References

[1] Ravnik J , Smigoc T , Bunc G , et al. Hypophyseal metastases: a report of three cases and literature review. Neurol Neurochir Pol. 2016;50:511‐516. doi:10.1016/j.pjnns.2016.08.007 27633123

[2] Murrone D , Abbate FA , Dpaulis D , Galzio R . Pituitary gland metastases from renal cell carcinoma: a case report and literature update. Integr Cancer Sci Therap. 2015;2:208‐210. doi:10.15761/ICST.1000142

[3] Kramer CK , Ferreira N , Silveiro SP , Gross JL , Dora JM , de Azevedo MJ . Pituitary gland metastasis from renal cell carcinoma presented as a non‐functioning macroadenoma. Arq Bras Endocrinol Metabol. 2010;54:498‐501. doi:10.1590/s0004-27302010000500011 20694412

[4] Ariel D , Sung H , Coghlan N , Dodd R , Gibbs I , Katznelson L . Clinical characteristics and pituitary dysfunction in patients with metastatic cancer to the Sella. Endocr Pract. 2013;19:914‐919. doi:10.4158/EP12407.OR 23757610

[5] Fassett DR , Couldwell WT . Metastases to the pituitary gland. Neurosurg Focus. 2004;16:E8. 15191337

[6] Gilard V , Alexandru C , Proust F , Derrey S , Hannequin P P , Langlois O . Pituitary metastasis: is there still a place for neurosurgical treatment? J Neurooncol. 2016;126:219‐224. doi:10.1007/s11060-015-1967-y 26514360

[7] Tosaka M , Higuchi T , Horiguchi K , et al. Preoperative evaluation of Sellar and Parasellar macrolesions by [(18) F] fluorodeoxyglucose positron emission tomography. World Neurosurg. 2017;103:591‐599. doi:10.1016/j.wneu.2017.04.032 28427982

[8] Melmed S , Casanueva FF , Hoffman AR , et al. Diagnosis and treatment of hyperprolactinemia: an Endocrine Society clinical practice guideline. J Clin Endocrinol Metab. 2011;96:273‐288. doi:10.1210/jc.2010-1692 21296991

[9] Stanisic TH , Donovan J . Prolactin secreting renal cell carcinoma. J Urol. 1986;136:85‐86. doi:10.1016/s0022-5347(17)44738-0 3712624

[10] Turkington RW . Ectopic production of prolactin. N Engl J Med. 1971;285:1455‐1458. doi:10.1056/NEJM197112232852604 5166245

[11] Basaria S , Westra WH , Brem H , Salvatori R . Metastatic renal cell carcinoma to the pituitary presenting with hyperprolactinemia. J Endocrinol Invest. 2004;27:471‐474. 15279082 10.1007/BF03345294

[12] Palapattu GS , Kristo B , Rajfer J . Paraneoplastic syndromes in urologic malignancy: the many faces of renal cell carcinoma. Rev Urol. 2002;4:163‐170. 16985675 PMC1475999

[13] Wendel C , Campitiello M , Plastino F , et al. Pituitary metastasis from renal cell carcinoma: description of a case report. Am J Case Rep. 2017;18(7–11):7‐11. doi:10.12659/AJCR.901032 28044054 PMC5223779

